# Lateral Diffusion on Tubular Membranes: Quantification of Measurements Bias

**DOI:** 10.1371/journal.pone.0025731

**Published:** 2011-09-29

**Authors:** Marianne Renner, Yegor Domanov, Fanny Sandrin, Ignacio Izeddin, Patricia Bassereau, Antoine Triller

**Affiliations:** 1 Institut de Biologie de l'Ecole Normale Supérieure (IBENS), Institut National de la Santé et de la Recherche Médicale U1024, Centre National de la Recherche Scientifique UMR8197, Paris, France; 2 Institut Curie, Centre de Recherche, Membrane and Cell Functions Group, Centre National de la Recherche Scientifique UMR168, Physico-Chimie Curie, Université Pierre et Marie Curie, Paris, France; 3 Laboratoire Kastler Brossel, Département de Physique, Institut de Biologie de l'Ecole Normale Supérieure (IBENS), Paris, France; The Research Center of Neurobiology-Neurophysiology of Marseille, France

## Abstract

Single Particle Tracking (SPT) is a powerful technique for the analysis of the lateral diffusion of the lipid and protein components of biological membranes. In neurons, SPT allows the study of the real-time dynamics of receptors for neurotransmitters that diffuse continuously in and out synapses. In the simplest case where the membrane is flat and is parallel to the focal plane of the microscope the analysis of diffusion from SPT data is relatively straightforward. However, in most biological samples the membranes are curved, which complicates analysis and may lead to erroneous conclusions as for the mode of lateral diffusion. Here we considered the case of lateral diffusion in tubular membranes, such as axons, dendrites or the neck of dendritic spines. Monte Carlo simulations allowed us to evaluate the error in diffusion coefficient (*D*) calculation if the curvature is not taken into account. The underestimation is determined by the diameter of the tubular surface, the frequency of image acquisition and the degree of mobility itself. We found that projected trajectories give estimates that are 25 to 50% lower than the real *D* in case of 2D-SPT over the tubular surface. The use of 3D-SPT improved the measurements if the frequency of image acquisition was fast enough in relation to the mobility of the molecules and the diameter of the tube. Nevertheless, the calculation of *D* from the components of displacements in the axis of the tubular structure gave accurate estimate of *D*, free of geometrical artefacts. We show the application of this approach to analyze the diffusion of a lipid on model tubular membranes and of a membrane-bound GFP on neurites from cultured rat hippocampal neurons.

## Introduction

The utilization of single-particle tracking (SPT) to study lateral diffusion and sorting of molecules in living cells has boosted these last years. In particular in neurons, this technique allowed the understanding of real-time dynamics of neurotransmitter receptors and other membrane molecules such as lipids or a membrane-bound GFP (GFP-GPI) [1], [2]. Neuronal synapses are established between neurites, which are tubular structures with diameters ranging from 50–300 nm (axons) up to more than 1000 nm (dendrites). All the factors that can regulate the diffusion of molecules such as corralling by sub-membranous fences, obstacles, molecular crowding and/or variations in membrane fluidity (reviewed in [3]) may influence synaptic transmission by affecting the equilibrium between extrasynaptic and synaptic receptors [4]. In addition to this, the plasma membrane of neurites exhibits a high curvature, which may impose restrictions to diffusion but also complicate the measurements. In non-planar surfaces, 2D SPT trajectories are the projections on a flat plane of the real displacements in the 3D surface. The use of Cartesian coordinates to quantify the displacements induces an underestimation of the mobility that is expected to depend on the diffusivity of the molecules, the membrane curvature and the frequency of image acquisition. In the case of cylindrical structures, polar coordinates should be used to quantify adequately the displacements transversal to the cylinder axis, whereas Cartesian coordinates are used for longitudinal displacements [5].

The influence of non-planarity on diffusion measurements has been previously addressed in case of measurements obtained by fluorescence recovery after photobleaching (FRAP) [6], [7], [8]. Theoretical calculations and analysis of simulated trajectories demonstrated that diffusion anisotropy can appear when the membrane is curved [5], [7] and the calculated diffusion constants can differ by a factor of ∼2 with the real ones [6], [8]. Here we evaluated the bias in diffusion measurements that appears in SPT (in 2D and 3D) on small tubular structures using standard image acquisition protocols. The effect of membrane curvature on the accuracy of diffusion measurements is difficult to address directly on cells due to the presence of numerous elements such as the cytoskeleton that may influence lateral diffusion. We made use of Monte Carlo simulations to reveal the interplay between tube size, diffusivity and sampling rate in their influence on SPT diffusion measurements. The observations done on simulated trajectories were validated by performing SPT of quantum dots (QD) on different probes that diffuse freely on artificial tubes or the surface of cultured hippocampal neurons. Artificial membrane tubes can be pulled from model membrane systems of controlled composition (Giant Unilamellar Vesicles, GUV) using micromanipulation and optical tweezers. This tube system has already been used to investigate the role of tube diameter on the lipid and protein distribution [9], [10], [11]. The radius of the tube can be adjusted by changing the membrane tension; therefore the diffusion of a given molecule on the same tube can be measured at different tube diameters.

We calculated a single dimensionless parameter that allows the estimation of the ratio between the real diffusivity and the one calculated on projected trajectories when the diameter of the tube is known. We also propose and compare different methods to measure the tube diameter using SPT data. Finally, we present a simple manner to overcome the geometrical bias by taking into account only the displacements in the direction of the tube axis, which provides the correct diffusion coefficient.

## Methods

### Ethics Statement

Rat primary hippocampal neurons were prepared in accordance with the guidelines issued by the French Ministry of Agriculture and approved by the Direction Départamentale des services Vétérinaires de Paris (Ecole Normale Supérieure, Animalerie des Rongeurs, license B 75-05-20). All efforts were made to minimize animal suffering and to reduce the number of animals used.

### Monte Carlo simulations

**Figure 1:**
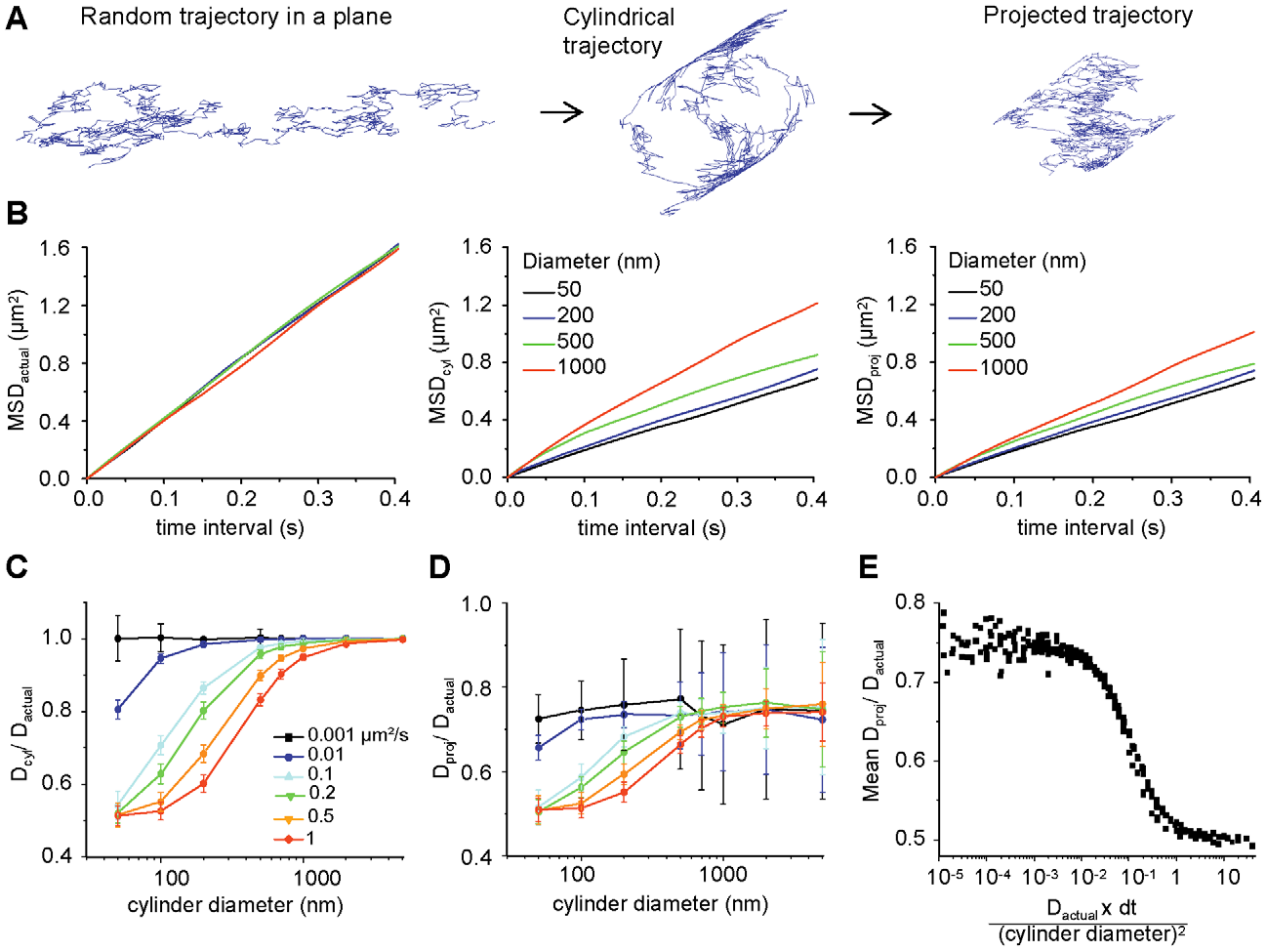
of geometry on diffusion measurements on cylindrical structures. *A*) Example of a random trajectory simulated on a plane and the derived cylindrical and projected trajectories. *B*) Examples of MSD plots of the original trajectories (*MSD_actual_, left*), the trajectories on cylindrical surfaces (*MSD_cyl_, centre*) or projected (*MSD_proj_, right*) ones, for cylinders of the indicated diameters. Trajectories were simulated with a diffusivity of 1 µm^2^/s. *C*–*D*) Ratios of *D* calculated on trajectories on cylindrical surfaces (*C*, *D_cyl_*) or projected (*D*, *D_proj_*) trajectories to the real diffusion constant of the original trajectory in the plane (*D_actual_*), as a function of the diameter of the cylinder. Each curve represents the mean ± SD values for 50 trajectories simulated to have the indicated diffusivities (0.001 to 1 µm^2^/s). *E*) The mean ratio *D_proj_ / D_actual_* as a function of the dimensionless parameter (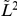) incorporating the diffusion coefficient (*D_actual_*), the image acquisition interval (*dt*) and the cylinder diameter (Ø).

Planar trajectories were simulated in two dimensions [12]. The *x* and *y* components of the *i*-th displacement step in the trajectory were randomly selected from two independent normal distributions with the mean of zero and the variance equal to 2*D_sim_dt*. Sets of 50 trajectories of 1000 points in length were simulated for each combination of *D_sim_* (0.001, 0.005, 0.01, 0.05, 0.1, 0.2, 0.5 or 1 µm^2^/s) and *dt* (5, 15, 30, 50, 75 or 100 ms), typical values of SPT experiments [13]. These planar trajectories were used to envelope cylinders with diameter *Ø* (50, 100, 200, 500, 700, 1000, 2000 or 5000 nm) and thus obtain trajectories on cylindrical surfaces (Fig. 1 *A* and Fig. S1 in Supporting Information). The axis of the cylinder was set parallel to the x-axis so that the coordinates in the x-axis remained unchanged. The positions around the cylinder, defined by the angle *θ* were found using *Δθ = Δy/(Ø/2)*. A randomly chosen angle was assigned to the first point. The new positions in the *y-* and *z-*axes were calculated as *y = Ø/2 cos θ* and *z = Ø/2 sin θ.* Finally, the projections of these trajectories on tubular surfaces were obtained by eliminating the z coordinate (Fig. 1 *A* and Fig. S1 in Supporting Information). Therefore, the diffusion calculations were performed on the same trajectory with three different geometries: planar, cylindrical and its projection on a plane.

### Artificial tubes

The giant unilamellar vesicles (GUVs) were prepared by electroformation on indium-tin oxide coated glass slides as described previously [14]. A mixture of porcine brain sphingomyelin and cholesterol at a 50∶50 molar ratio, complemented with 0.01% of 1,2-distearoyl-*sn*-glycero-3-phosphoethanolamine-N-[biotinyl(polyethylene-glycol)-2000] (DSPE-PEG(2000) Biotin), was used to prepare artificial membranes. All lipids were from Avanti Polar Lipids (Alabaster, AL, USA). Electroformation was carried out for 3 hours in sucrose solution with osmolarity of 200 mOsm at 60°C and the final AC voltage on the ITO slides of 900 mV at 12 Hz. The GUVs were then diluted with a buffer with matching osmolarity (202–205 mOsm) containing 20 mM Hepes, 50 mM NaCl, ca. 75 mM glucose and 40 mg/L casein at pH 7.0 and a small amount (ca. 10 fmol) of QD625-streptavidin conjugate (Invitrogen, Cergy Pontoise, France) was added. The GUVs were then washed with the buffer in a centrifuge 3 times for 1 minute at 1000g. Labeled GUVs were transferred to the microscopy observation chamber pretreated with casein (1 g/L) and aspirated in a glass micropipette using a micromanipulator (Narishige, Tokyo, Japan) and a custom-made hydraulic system. The membrane tension was controlled by changing the aspirating hydrostatic pressure in the micropipette. The bilayer tube (tether) was pulled from the GUV with a streptavidin-coated polystyrene bead (Spherotech, Lake Forest, IL, USA) held with a custom-made fixed optical trap (see [9] for details). The pulling force was deduced from the bead displacement in the trap, the stiffness of which was calibrated beforehand.

### Single particle imaging on artificial tubes

The high-speed imaging of single QDs attached to lipid molecules in the artificial membrane tubes was made using an epi-fluorescence microscope (eclipse T*i*, Nikon France SAS, Champigny-sur-Marne, France) equipped with a high-pressure Hg lamp as a light source and a back-thinned EMCCD camera (iXon DU-897, Andor Technology, Belfast, Ireland). The CCD physical pixel size was 16×16 µm (pixel size on the image: 160 nm). The measurements were performed using a Nikon Plan Fluor 100x oil-immersion objective with numerical aperture of 1.3. Fluorescence filter set QD625 (BP435/40, dichroic 510 nm, BP625/15) was obtained from Semrock (Rochester, NY, USA). For each tube at a given diameter a sequence of 500 or 1000 images was obtained with 15-ms exposure time (time between consecutive frames ca. 15.7 ms). In a typical experiment we would have between 3–10 individual QD on a membrane tube of 20–50 µm in length.

### Cell culture and transfection

Hippocampal neurons from 18-day-old Sprague-Dawley rat embryos were cultured at a density of 6x10^4^ cells/cm^2^ on coverslips pre-coated with 80 µg/ml poly-D,L-ornithine (Sigma Aldrich, Lyon, France) and 5% fetal calf serum (Invitrogen, Cergy Pontoise, France). Cultures were maintained in serum-free neurobasal medium supplemented with B27 (1X) and glutamine (2 mM) (Invitrogen, Cergy Pontoise, France). Neurons were transfected at 7 days in vitro (DIV) using Lipofectamine2000 (Invitrogen, Cergy Pontoise, France). GFP-GPI plasmid was kindly provided by Dr. S. Mayor [15].

### Single particle imaging of GFP-GPI

For SPT of GFP-GPI, QDs emitting at 605 nm conjugated with goat F(ab')2 anti-rabbit IgG (Invitrogen, Cergy Pontoise, France) were previously coupled with an anti-GFP antibody (rabbit polyclonal, Synaptic Systems, Goettingen, Germany) as in [2]. Briefly, QDs (30 nM) were incubated first with the antibody (5 nM, 30 min) and then for an additional 15 min with casein. Cells were incubated for 10 min with the pre-coupled QDs (0.06 nM) and rinsed. All incubation steps and washes were performed at 37°C in MEM recording medium (MEMr: phenol red-free MEM, glucose 33 mM, HEPES 20 mM, glutamine 2 mM, Na-pyruvate 1 mM, and B27 1X). Within 30 min after QD staining, cells were imaged in the MEMr at 37°C in an open chamber using an inverted microscope (IX70, Olympus France, Paris, France) equipped with a 60X objective (NA 1.45; Olympus France, Paris, France). Fluorescence was detected using a xenon lamp, appropriate filters (QD: D455/70x, HQ605/20m; dichroic E590lpv2; GFP: HQ500/20, HQ535/30m; Chroma Technology, Roper Scientific, Evry, France) and a CCD camera (Cascade 512BFT, Roper Scientific, Evry, France). In this set up the CCD physical detector had 16×16 µm with a pixel size of 167 nm. QDs were recorded during 1000 consecutive frames at 66 Hz with continuous illumination.

To obtain information of the position of particles on the Z-axis we introduced a weak cylindrical lens into the optical detection path [16], [17]. In these conditions, the shape of the point spread function of the imaged QDs is circular in the plane of focus but ellipsoidal above and below focus therefore the position in the Z-axis can then be extracted from the image shape and orientation (Fig. S2 *A* in Supporting Information). In this case, the frequency of acquisition was 33 Hz.

### Tracking and quantitative analysis

Single QDs were identified by their blinking. Tracking was performed with homemade software in MATLAB (The Mathworks, Natick, MA, USA). Fluorescent peaks in each image frame of the movie were identified by fitting local maxima with a Gaussian function corresponding to the point spread function of the experimental set up. This allowed deducing the peak intensity and the centroid position in the two lateral dimensions with a localization (pointing) accuracy of ∼10 nm. The localization accuracy was determined by tracking QDs immobilized on a coverslip. When applicable, the position in Z was retrieved by a second fit to an elliptical Gaussian function to deduce the width of the peak in the two lateral dimensions, *w_x_* and *w_y_*. The ratio *w_x_*/*w_y_* was used to find Z by interpolation, using a previously generated calibration curve (Fig. S2 *B* in Supporting Information). The calibration curve was determined using 100 nm-diameter fluorescent beads dried on a coverslip. The localization accuracy in Z-axis was determined as the dispersion in Z calculated on QDs dried on a coverslip. We could determine the Z position in a ∼400 nm range with ∼50–70 nm of localization accuracy. The spots in a given frame were associated with the maximum likely trajectories estimated on previous frames of the image sequence. We discarded trajectories with less than 100 points in case of GFP-GPI, or 30 points in case of artificial tubes. Trajectories had on average 549 points in 2D SPT of GFP-GPI, 685 points in 3D SPT of GFP-GPI and 73 points for artificial tubes. The mean-square displacement (MSD) was calculated using *MSD*(*ndt*) = (*N-n*)^−1^∑_i = 1_^N-n^((*x_i+n_- x_i_*)^2^ +((*y_i+n_- y_i_*)^2^), where *x_i_* and *y_i_* are the coordinates of an object on frame *i*, *N* is the total number of steps in the trajectory, *dt* is the time interval between two successive frames and *ndt* is the time interval over which displacement is averaged [17]. One-dimensional MSD was calculated taking into account the displacement in only one dimension. The diffusion coefficient D was calculated by fitting the points 2 to 5 of the MSD plot versus time with the equations *MSD*(*t*) = 4*Dt* + *b* (two dimensions) or *MSD*(*t*) = 2*Dt* + *b* (one dimension). The offset *b* includes both static and dynamic errors and thus it can be positive or negative [12], [18], [19]. Given the localization accuracy, trajectories with *D*<10^−4^ µm^2^/s were considered as being immobile. Images were prepared using Photoshop (Adobe Systems, Paris, France).

## Results and Discussion

Lateral diffusion along a membrane is not, in principle, affected by membrane geometry unless the curvature modifies molecular interactions, as it could be the case for very high curvature. Therefore, changes in diffusion may indicate the presence of particular interactions. However, the technical limitations of the different approaches to measure diffusion on curved surfaces preclude this simple conclusion. The diffusion on tubular structures has been deduced from Monte Carlo simulations to evaluate the influence of the tube diameter and the experimental conditions on the quantification of mobility. We have then compared simulated data with experiments on artificial membrane tubes and on neurites using 2D and 3D SPT.

### Bias introduced by geometry in diffusion measurements on simulated trajectories

Trajectories were first constructed on a flat plane using diffusivities similar to those of membrane molecules on neurites [13]. The time between trajectory points (*dt*) was in the usual range of SPT image acquisition frequencies (5–100 ms). The planar trajectories were then used to envelope cylinders of different diameters (50–5000 nm) and the obtained trajectories around cylindrical surfaces (cylindrical trajectories, the 3D-SPT outcome) were finally projected onto a plane, simulating the 2D SPT outcome (Fig. 1 *A* and Fig. S1 in Supporting Information). Thus, we were able to compare the mean-squared displacement (MSD) and the diffusion coefficient (*D*, proportional to the initial slope of the MSD curve) calculated on the same trajectory in the three situations.

In a system with free Brownian diffusion we expect to see no effect of tube radius on diffusion for radii wider than 100 nm. Some corrections due to hydrodynamic effects are expected for narrower tubular structures [20]. At the same time, the displacements in the direction normal to the tube axis should be systematically underestimated due to the fact that 1) by the projection of each step the distance in the z-axis is lost, and 2) the displacement along the perimeter of a circumference is measured as the span and not the arc of the corresponding angle. Fig. 1 *B* shows examples of the MSD of trajectories that enveloped cylinders of different diameters using *D* = 1 µm^2^/s and *dt* = 15 ms. The MSD was affected both in the cylindrical and in the projected trajectories, the slope being smaller than the real one (Fig. 1 *B*). *D* was calculated on the original trajectories (*D_actual_*), the cylindrical trajectories (*D_cyl_*) and the projected (*D_proj_*) ones. The ratio of *D_cyl_* or *D_proj_* over *D_actual_* varied between ∼0.5 to ∼1 depending on the diffusivity of the molecules and the diameter of the cylinder (Fig. 1 *C* and *D*). The underestimation of D was maximal in case of high diffusivity and small diameters. In the case of cylindrical trajectories the difference eventually disappeared as the diameter increased, (Fig. 1 *C*) but in the case of projected trajectories, *D_proj_* was always inferior to *D_actual_* levelling off at ∼0.75 *D_actual_* (Fig. 1 *D*). This means that the error due to the difference between the span and the arc dominated on small cylinders, whereas the error due to the loss of the displacements in z dominated on larger ones. As the error that appeared on *D_cyl_* “propagated” to *D_proj_*, the ratio *D_proj_*/*D_cyl_* was close to 1 for thin cylinders and high diffusivities, but it decreased to ∼0.75 in larger cylinders (Fig. S3A in Supporting Information). Note that the ratio *D_proj_*/ *D_actual_* (Fig. 1 *D*) had a large dispersion of values that was more pronounced for larger tubes and for low diffusivities, because the limited number and length of simulated trajectories made the simulation very sensitive to the random choice of the initial particle position on the tube.

The difference between *D_cyl_* or *D_proj_* and *D_actual_* also depended on *dt*, which influences the reliability in the measure of the displacements on a curved surface (Fig. S3C-E in Supporting Information). This underestimation was larger at longer *dt* (lower acquisition frequency) and, as expected, the *D_cyl_* was more affected than *D_proj_* (Fig. S3C-E in Supporting Information). For example, using a *dt* of 100ms, it was not possible to calculate a *D_cyl_* equal to *D_actual_* in cylinders of less than 200 nm in diameter even in case of very slow diffusivity (Fig. S3E1 in Supporting Information).

The degree of underestimation of the diffusion coefficients calculated from the projected trajectories could be estimated calculating a single dimensionless parameter 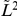 which includes the experimental conditions together with the diffusivity of the molecule: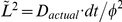, where 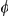 is the diameter of the cylinder. This parameter 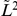 is in fact the square of the ratio of the mean distance travelled by a particle between two consecutive acquisitions to the cylinder diameter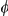. Fig. 1 *E* summarizes the results for *D_proj_*, obtained from simulations done in a wide range of *D_actual_* (0.001–1 µm^2^/s), *dt* (5–100 ms) and cylinder diameters (50–1000 nm) that one can encounter in the SPT experiments with biological membranes. When the distance between two consecutive points in a trajectory is at least two orders of magnitude smaller than the cylinder diameter, the diffusion coefficient is underestimated by 25%, whereas for longer distances the underestimation reaches up to 50%. The differences obtained here between the actual and the measured diffusivities are similar to those that were previously reported for FRAP (fluorescence recovery after photobleaching) studies [6], [8]. The parameter 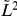 was also calculated for *D_cyl_* (Fig. S3B in Supporting Information). In this case the underestimation ranged from ∼0% to ∼50%. Fig. 1 *E* and S3B provide a practical tool to estimate the error in diffusion coefficient calculation for given experimental conditions, as long as the diameter of tubular surface is known.

**Figure 2:**
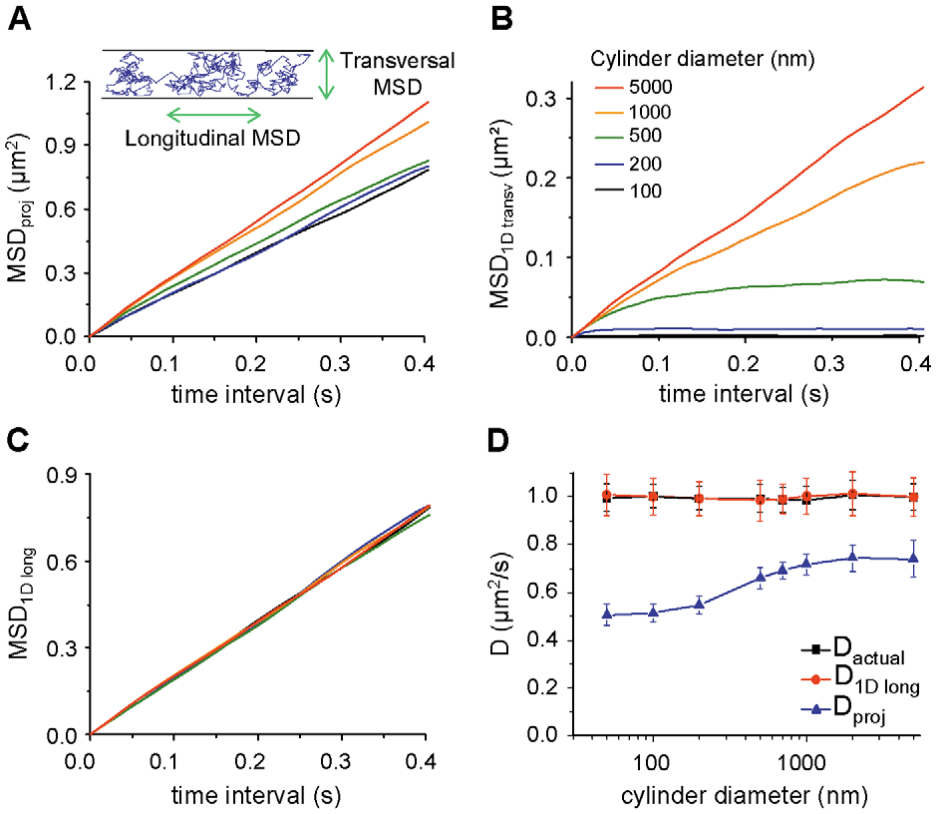
Unbiased D calculation using the displacements in the direction of the cylinder axis. *A*–*C*) Examples of MSD plots of projected trajectories (*A*) and their corresponding *MSD_1Dtransv_* (*B*) and *MSD_1Dlong_* (*C*) for the indicated cylinder diameters. Trajectories were simulated with a diffusivity of 1 µm^2^/s. Inset in A: the projection of each simulated trajectory was decomposed into two components: parallel and perpendicular to the cylinder axis. These components were used to calculate the longitudinal (*MSD_1Dlong_*) and transversal (*MSD_1Dtransv_*) MSD. *D*) Effect of cylinder diameter on the values of *D_actual_*, *D_proj_* or *D* calculated on *MSD_1Dtransv_* (*D_1Dlong_*), for trajectories simulated with a diffusivity of 1 µm^2^/s (mean ± SD, n = 50).

For tubular surfaces, the components of the movement that are parallel to the tube axis direction (longitudinal steps) do not depend on the curvature of the surface, whereas transversal steps have a maximum possible size that is the diameter of the tube. A separate analysis of the longitudinal and transversal components allows splitting physical and geometrical effects associated with the curvature of the membrane surface [5], [21]. The MSD were calculated for projected trajectories as well as for their transversal (*MSD_1Dtransv_*) and longitudinal (*MSD_1Dlong_*) components (Fig. 2 *A*–*C*). This a particular case of the spline analysis recently proposed by Long and Vu [21]. As expected, *MSD_1Dtransv_* plot reached the asymptote related to the cylinder diameter (Fig. 2 *B*) and the *MSD_1Dlong_* was not affected by size of the cylinder (Fig. 2 *C*). We calculated the corresponding *D* from *MSD_1Dlong_* (*D_1Dlong_*), which was similar to *D_real_* for all cylinder diameters (Fig. 2 *D*). These analyses were then applied to experimental SPT data obtained on artificial membrane tubes of controlled diameters and on neurites of living neurons.

### Diffusion analysis on artificial tubes and tube diameter measurement

**Figure 3:**
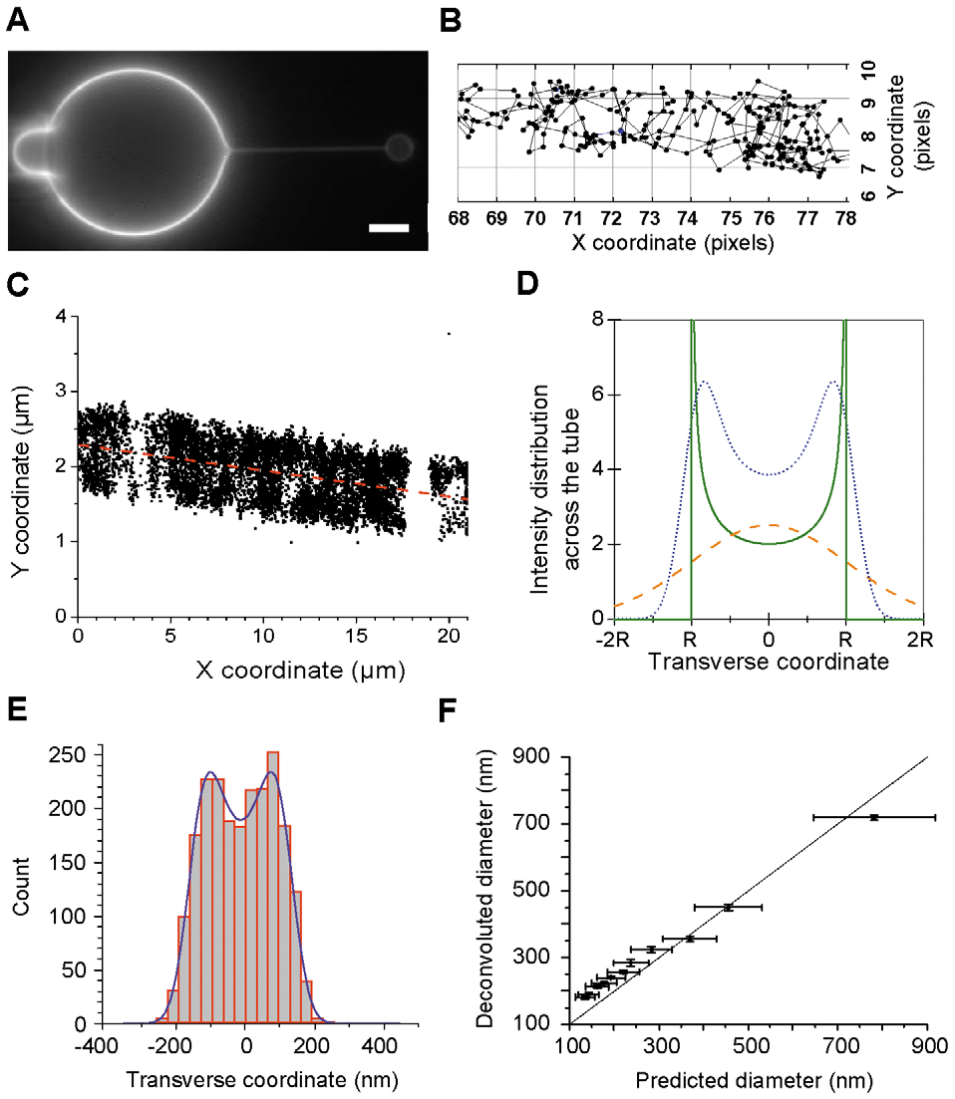
SPT allows simultaneous measurement of diffusion and tube diameters. *A*) The model system where a thin tubular tether is pulled by an optically trapped, streptavidin-coated bead (*right*) from a giant unilamellar vesicle (GUV) tagged with biotinylated lipids. Changing the aspiration pressure in the micropipette (*left*) holding the GUV allows us to vary and control the diameter of the tether. Scale bar: 5 µm. *B*) An example of connected trajectory obtained on a membrane tube. Several tens of trajectories were constructed on every tube for the MSD analysis. One pixel is 160 nm. *C*) An example of the QD positions extracted from a series of 1000 images (before trajectory reconnection). The direction of the tube is determined by a linear fit of all detected positions (*red dashed line*). For the analysis of the transverse distribution of the QD positions, the coordinate plane is further rotated (*not shown*) to align the X axis with the direction of the tube. *D*) Mathematically predicted profile for the projection of points uniformly distributed over the surface of a tube on a plane (*green solid line*, cross-section is shown, Eq. 1). A Gaussian profile with the same area is shown for comparison (*orange dashed line*, Eq. 2) as well as the result of convolution (*blue dotted line*) of the predicted profile with a Gaussian according to Eq. 3. *E*) An example of the transverse distribution of the QD positions extracted from the series of images. The distribution is fit by the convolution (*violet line*) of the tube projection profile and a Gaussian (see panel *D*) to extract the tube diameter and the smearing parameter (see text). *F*) Comparison of the tube diameters obtained from the analysis of transverse distribution of the QD positions and the diameters calculated based on the membrane tension and the pulling force (mean ± SD).

Giant unilamellar vesicles (GUV) were prepared using a mixture of purified lipids, cholesterol and sphingomyelin, at a 1:1 molar ratio. This lipid composition was chosen to ensure high bending rigidity of the bilayer and, hence, relatively large radius of the resulting tubes. In addition, this composition gives rise to relatively low diffusion coefficients of ∼0.25 µm^2^/s, which is in the range to the values of lipid diffusion measured on live neurons [2]. Bilayer tubes were pulled from a GUV using a streptavidin coated polystyrene bead held by optical tweezers (Fig. 3 *A*). The diameter of the bilayer tube could be changed and controlled by adjusting the aspiration pressure in the micropipette holding the GUV and thus changing the membrane tension [22]. Streptavidin-coated QDs were attached to the membranes by adding a trace amount (0.01 mol%) of synthetic lipid carrying a biotin group on a flexible linker (PEG-2000). The labelling was performed at low concentration to allow the detection and tracking of individual molecules (Movie S1). QD trajectories could be obtained on the tubes (Fig. 3 *B*), similarly to what was previously made on living neuronal structures [2]. As the free area theory predicts that the diffusivity should increase when the membrane is stretched to effectively increase the area of “voids” between lipids [23], we have measured *D* on the surface of GUVs for different membrane tensions. For membrane tensions 10^−6^ – 10^−4^ N/m), covering the range of values used in our tube experiments (10^−6^– 4·10^−5^ N/m), no statistically significant change of *D* was found (data not shown). Therefore, much higher tensions than the ones used here might be necessary for a noticeable effect on the diffusion. Therefore the effects that we measure in our tube experiments are only related to the geometry and not to the membrane tension.

As many tubular structures in cells have diameters smaller than the diffraction limit of conventional microscopy a super-resolution imaging technique is required to determine accurately the curvature of the surface. The SPT technique used here for diffusion measurements is in fact equally well suited for determination of the size (and shape) of tubular membranes below the limit of optical resolution. We have first tested this approach with artificial membrane tubes, where the diameter can be estimated independently of the SPT technique. Taking advantage of the single-molecule pointing accuracy of SPT we re-constructed the shape of the tubes with ∼20 nm resolution using all the successive positions of QDs in an image series (Fig. 3 *C*). As a result, we were able to directly estimate the diameter of the tube using the transverse distribution of the QD positions and appropriate fitting. The direction of the tube was first determined by linear regression of all detected positions (Fig. 3 *C*, red dashed line). Then the coordinate plane was rotated to align the X axis with the direction of the tube and the transverse distribution of the QD positions (Y coordinates obtained after rotation) along the whole tube was fitted with a theoretically predicted distribution.

To model the transverse distribution we used the convolution of the projection of a tube on a plane (Fig. 3 *D*, green line) with a Gaussian profile (orange line). The mathematically predicted profile for the projection of points uniformly distributed over the surface of a tube on a plane is expected for an ideal straight tube with constant diameter. It predicts higher probability to find a fluorescent marker closer to the side of the tube than in the middle, because when moving around a cylinder the projected position varies much more slowly close to the edge of a cylinder than on its top. The convolution with a Gaussian was introduced (as a first approximation) to incorporate the effects of finite localization accuracy, small deviations from ideally cylindrical shape, the variation of the tube diameter along the tube length and with time, and fluctuations of the tube position as a whole [24]. Therefore, for a tube of diameter 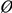 centred at the transverse coordinate *y_c_* the projection profile is



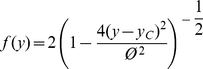
(1)A Gaussian distribution with a width *w* corresponds to
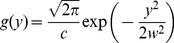
(2)The convolution 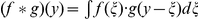 of the two profiles (with a substitution 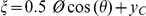 to avoid singularities at ± 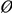/2) gives
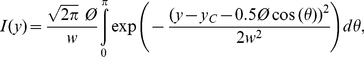
(3)which we numerically integrated using MATLAB.

Fitting the experimental distributions with equation (3) allowed us to extract the tube diameter *Ø* and the “smearing” parameter *w* (Fig. 3 *E*). For example, fitting the distribution of QD positions shown in Fig. 3 *E* yields a tube diameter *Ø* = 225±10 nm and a smearing value *w* = 45±6 nm.

In the case of artificial tubes pulled by aspiration/micromanipulation technique, the tube diameter *Ø* can be calculated based on mechanical considerations when the membrane tension σ and the pulling force *f* are known [9]: *Ø* = *f/ (2πσ)*. Using this relationship we were able to validate our method for determining the diameter of the tubular membrane from the SPT data. Fig. 3 *F* compares the diameters of the artificial tubes determined by the two methods. The values agree well with minor deviations that can be attributed to the limited precision of the calculation of the membrane tension that determines the predicted diameter (note the error bars).

**Figure 4:**
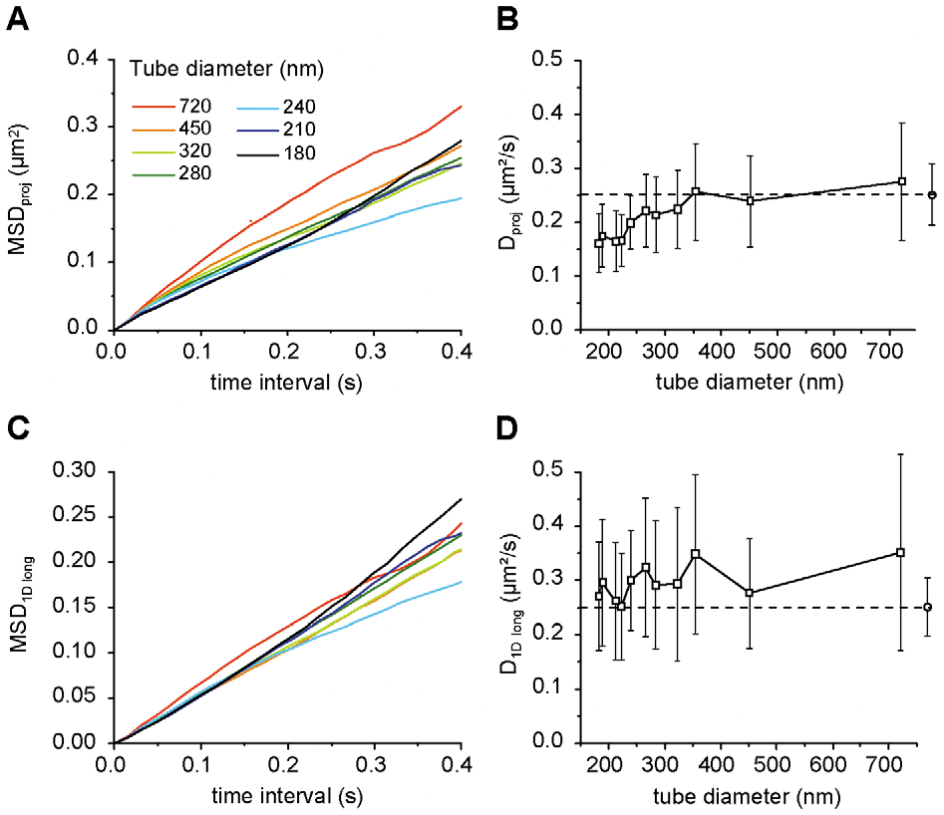
Analysis of diffusion on artificial tubes. *A,C)* Averaged MSD plots of projected 2D-trajectories (*A*) and of the longitudinal components (*C*) of the displacements. The diameter of the tubes varied between the indicated values. *B,D)* D calculated from the corresponding MSD plots on the left. The reference value of the diffusion coefficient measured on the GUV surface (quasi-planar membrane) is shown with dashed horizontal lines and open circles (mean ± SD).

Diffusion on artificial tubes was measured for the same QDs while varying the diameter of the tube between 150 and 700 nm. Similarly to what was observed on simulated trajectories, the initial slope of the MSD curves increased with the tube diameter (Fig. 4 *A*). Some curves did not show significant deviation from a linear dependence and some were slightly curved, which could be otherwise attributed to the experimental variability if the effect of curvature was not taken into account. From these MSDs, we have deduced the diffusivities and plotted them as a function of the tube diameter (right column in Fig. 4). Fig. 4 *B* suggests an apparent decrease of the diffusion constant for thinner tubes pulled from the same GUV. The analysis on the transversal components of the displacements revealed that all of the MSD curves contained clearly non-linear components with the plateau levels depending on the tube diameter (not shown), which was not the case for the *MSD_1Dlong_* (Fig. 4 *C*). Consequently, *D_1Dlong_* was independent of the tube diameter (Fig. 4 *D*). All the values of *D_1Dlong_* agree, within the experimental error, with the diffusion coefficient measured on the GUV bottom surface, which can be considered nearly planar (dashed horizontal lines and open circles in Fig. 4 *B,D*). This shows that the diffusion was not affected by the membrane curvature in this diameter range.

An alternative method to measure the tube diameter is to use the *MSD_1Dtransv_* data as was proposed by Wieser and coworkers [5]. These authors have shown that the plateau in the transversal MSD plots corresponds to the square of the tube radius. Here, we could compare the plateau value with the actual tube diameter. The diameter corresponding to the plateau is in general larger than the actual value (Fig. S4 *A* in Supporting Information). The discrepancy might come from the fluctuations of the tube position and diameter; deviations from ideally cylindrical shape and limited localization accuracy, all of which make the plateau appear slightly higher than *Ø ^2^/4*.

### Diffusion of GFP-GPI on neurites

An experimental situation in which SPT is classically performed on tubular structures is the study of lateral diffusion on neurites. In this case, we and others have obtained very broad distributions of D with local heterogeneities [1], [2]. This may affect the bias due to the geometry in which molecules diffuse. For fast-diffusion molecules like lipids, the observed median D is in the order of 10^−1^ µm^2^/s on neurites, with D ranging from 10^−4^ to 10^0^ [2]. For the median values, simulations predicted a ratio of *D_proj_*/*D_actual_ of* ∼0.55–0.6 for cylinders of 100 nm and a ratio of *D_proj_*/*D_actual_ of* 0.65∼0.75 for cylinders of 200–500 nm in diameter (Fig. 1D). We wanted to check these values as well as the feasibility of the decomposition of the displacements that we proposed on cellular tubes.

**Figure 5:**
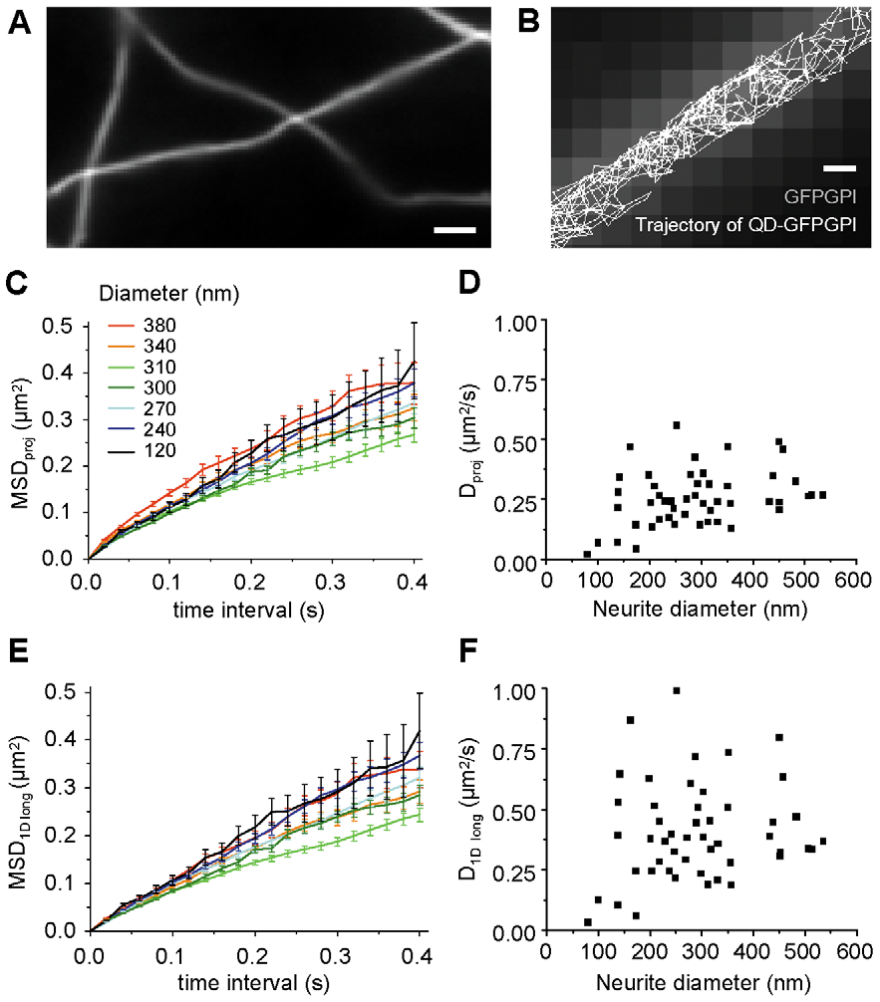
GFP-GPI diffusion on neurites. *A*) Neurites (axons) of hippocampal neurons transfected with GFP-GPI. Bar: 1 µm. *B*) A representative trajectory of GFP-GPI labeled with a QD on a neurite (white line). Epifluorescence image of GFP is shown in the background. Bar: 200 nm. *C,E,)* Examples of MSD of projected trajectories (*C*) and of longitudinal components (*E*) of the displacements for trajectories on neurites of the indicated diameters (mean ± errors calculated as in ref. [18]). *D,F)* The diffusion coefficients *D_proj_ (D)* and *D_1Dlong_ (F)* of GFP-GPI trajectories on neurites of different diameters. Pearson's correlation coefficients between the diffusion coefficients and diameter were 0.32667 (*D_proj_*), and 0.13832 (*D_1Dlong_*) (n = 49 trajectories on different neurites).

Cultured hippocampal neurons were transfected with a membrane bound GFP (GFP-GPI) and GFP-GPI-bound QDs were tracked as described before [2] (Fig. 5 *A,B*). Recordings were made on 75–550 nm wide neurites of young neurons (9–14 days after plating) far away from the cell body. Due to the glycosyl-phosphatidylinositol anchor (GPI), GFP-GPI is expressed on the outer leaflet of the plasma membrane, where it can be labelled with QDs conjugated to anti-GFP antibody (Movie S2). The GPI anchor favour the partition of GFP-GPI to lipid rafts, but this does not hinder its diffusion at the plasma membrane of neurons [2]. The diameter of the neurite was measured using the convolution method described for artificial tubes (Fig. S4 *B*). This method was successfully applied on straight stretches of neurites when trajectories cover sufficiently the surface. If the amount of trajectory points was not enough to obtain a good fit to equation (3), the shape of the neurite was reconstructed as a rectangle from the positions of QDs and the diameter was measured as the width of the rectangle (Fig. S4 *C*). When both methods could be applied, they provided similar results (Fig. S4 *B* and *C*). Thus, analysis of SPT trajectories can provide a reliable estimate of the diameter of tubular structures below optical resolution limit both on artificial and cellular tubular structures. However, since this method is based on fitting the distribution of stochastic data, a large enough number of particle positions (at least several thousands) is necessary for reliable fit. Therefore, when this method is applied to tubular membranes *in vivo*, a long enough straight section is necessary. We also extracted the diameter from the MSD plateau [5], which provided values comparable to the other methods (Fig. S4 *D*).

Similarly to simulations and trajectories on artificial tubes, we analyzed the MSD of the SPT trajectories and their *MSD_1Dlong_*. We selected straight portions of neurites to simplify the calculations, but the analysis of displacements in the longitudinal axis of the tube could be done on curved neurites using the spline analysis recently proposed by Long and Vu [21].

Figs. 5 *C* and *E* show the MSD of some trajectories on neurites and Figs. 5 *D* and *F* show the calculated diffusion coefficients of all the trajectories analyzed. The ratio *D_proj_*/*D_1Dlong_* varied between 0.52 and 0.82 (not shown). Whereas *D_proj_* ranged between 0.02 and 0.56 µm^2^/s, *D_1Dlong_* values were between 0.03 and 1 µm^2^/s (the actual *D* of GFP-GPI on these neurites). As we recently reported [25], the smaller values of D were found on the thinner neurites (Fig. 5 *D* and *F)*. This could be the consequence of the logarithmic dependence of the protein diffusion coefficient on the “membrane size” for membranes of finite size [25], but this could also reflect differences in membrane composition between thinner (axons) and wider (dendrites) neurites. We then tested the predicted error calculated using the dimensionless parameter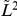 calculated using *D_proj_* (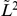 = 0.014 – 0.26) or *D_1Dlong_* (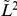 = 0.019 – 0.5). In both cases, the estimated ratio *D_proj_*/*D_1Dlong_* varied between ∼0.5–0.63, which was a good estimate of the observed ratio. It has to be noted that some neurites may flatten when they adhere to the coverslip which may introduce differences between the observed and the calculated ratio. Nevertheless, we could approximate the error in *D* calculation using 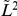 and the experimental values *D_proj_* or *D_1Dlong_*.

**Figure 6:**
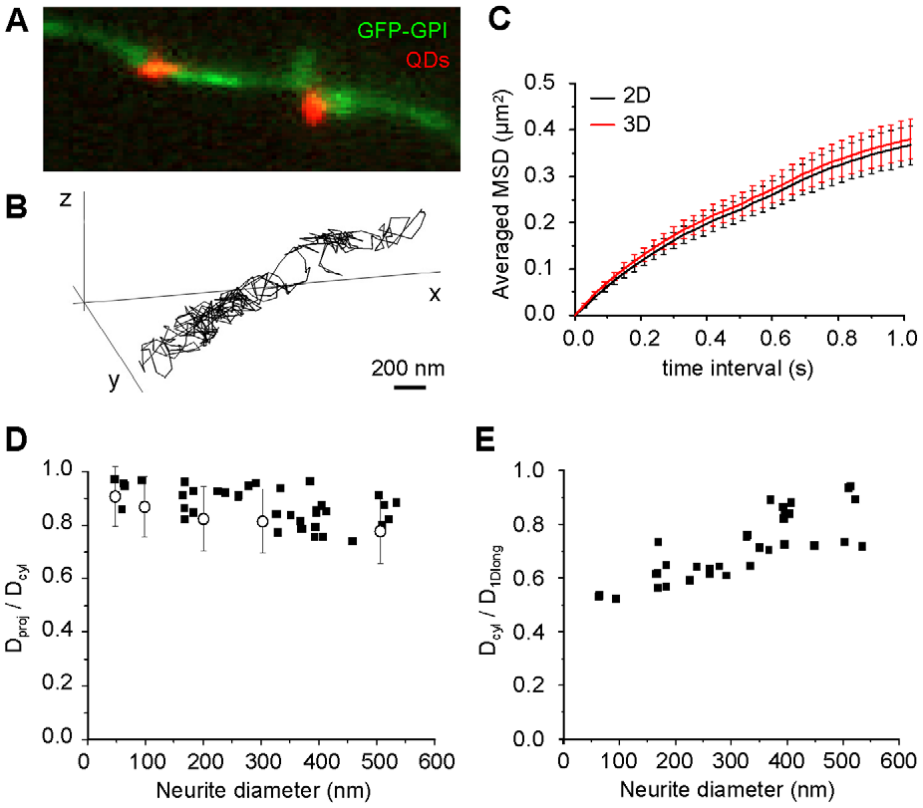
3D single particle tracking of GFP-GPI. *A*) Fluorescence image of a portion of neurite of a GFP-GPI transfected neuron (*green*) overlaid with two GFP-bound quantum dots (GFPGPI-QD, *orange*). The shape of the QD depends on its position in the Z axis. *B*) Example of a 3D GFPGPI-QD trajectory. Bar: 200 nm. *C*) averaged MSD plot for trajectories obtained by 3D SPT (3D, *red*) and their projections in the plane (2D, *black*) (mean ± sem) (n = 26 trajectories on different axons) *D*) Ratio of *D* on neurites (black squares) calculated without (*D_proj_*) or with (*D_cyl_*) the displacements in Z (n = 26 trajectories on different axons), and the equivalent ratio of *D* of simulated trajectories with diffusivities between 0.001 and 1 µm^2^/s (*circles*, mean ± s.e.m., n = 200) versus the diameter of the neurites or cylinders. *E*) Ratio *D_cyl_/D_proj_* versus the diameter calculated on the same neurites as D.

To get more precise estimates of the diffusion coefficients on curved membrane, a tempting technique to overcome the bias related to planar projection is 3D-SPT. As mentioned from our Monte Carlo simulations, the benefit of detecting 3D trajectories should be limited due to finite acquisition frequency. As indicated by simulations, even with a *dt* of 5 ms there is a important underestimation of tha actual D when the diffusion is high. We analyzed the diffusion of GFP-GPI on neurites with 3D-SPT introducing a cylindrical lens into the optical detection path of the experimental set-up. In these conditions, the shape of the point spread function of the QDs is circular in the plane of focus but ellipsoidal above and below focus; therefore the position in the Z-axis can then be extracted from the image shape and orientation [16], [17] (Fig. 6 *A*, Fig. S2 *A* in Supporting Information). Fluorescent peaks in each frame were fit to an elliptical Gaussian function to deduce the width of the peak in the two lateral dimensions, *w_x_* and *w_y_*. The ratio *w_x_*/*w_y_* was used to retrieve the position in z using a previously generated calibration curve (Fig. S2 *A* and *B*). X and Y coordinates were determined as for 2D SPT (Fig. 6 *B*).

By comparing the 3D trajectories and their projections to the X-Y plane on the same neurites, we could conclude that the MSD plots were similar (Fig. 6 *C*). Two diffusion coefficients D were measured for each trajectory, with (*D_proj_*) or without (*D_cyl_*) planar projection. As expected, *D_cyl_* values were larger than the corresponding *D_proj_* values, due to the loss of the displacements in z in the projection. The ratio between *D_proj_* and *D_cyl_* tended to decrease on larger neurites ranging between 0.99 and 0.74 (Fig. 6 *D*). The ratios obtained on simulated trajectories for equivalent *D* and cylinder diameters were not significantly different from those derived from experimental trajectories (Fig 6 *D*). This indicates that 3D SPT improved the calculation of *D*. However, the calculation of 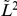 for the corresponding *dt* predicted an underestimation of *D* (ratio *D_cyl_*/*D_actual_*) of up to ∼0.5-0.55. Indeed, the ratio *D_cyl_*/*D_1Dlong_* was dependent on the diameter and is around 0.5 for the thinnest neurites (Fig. 6 *E*). Therefore the improvement achieved by using 3D SPT was not substantial in these conditions of image acquisition (33 Hz in our experimental set up).

### Conclusions

In SPT experiments, values of the diffusion coefficients on tubular membranes, usually deduced from projected trajectories, are underestimations of the real values by 25 to 50%. In addition, misleading conclusions can arise from the analysis of MSD and of Brownian movements because of the restricted transverse diffusion in narrow tubes. This problem can be overcome when extracting the component of the particle displacement longitudinal to the tube axis, a particular case of the analysis of displacements in the direction of a spline line [21]. If this is not possible, we have provided a practical way to estimate the error on *D* using Fig.1 *E*. We have validated this method on artificial tubes and on neurites. Single particle tracking measurements using QD-labelled lipids in artificial tubes on one hand, and GPI-anchored GFP conjugated to QDs in rat hippocampal neurons on the other hand, gave similar MSD dependences on membranes with different curvature. On artificial systems, we have showed that the longitudinal diffusivity on the tube is equal to the diffusivity on the non-curved GUV, independently of the tube diameter at least down to 150 nm, as expected from the simulations. This implies that this approach is also valid for neurites. It provides a means to compare the protein or lipid diffusion in different areas of a neuron, irrespectively of its own geometry, or compare neurons in different conditions. In particular, dendritic spines are membrane protrusions containing the sites of excitatory neurotransmission in hippocampal neurons (refs in [26]). Spine heads, which are roughly spherical structures (not analyzed here) are connected to the dendritic shaft (diameter >500 nm) by a thin neck (diameter <100 nm) which can be a few µm long [26]. It has been proposed that the spine neck could act as a barrier for the diffusing molecules [27]. We showed here that the analysis of diffusion on these thin tubes using SPT can only be done calculating D from the displacement parallel to the tube axis. Alternatively, the bias can be reduced by correctly adjusting the image acquisition frequency depending on the mobility of the molecule and on the tube diameter. In particular, we have also compared projected diffusivity with measurements from 3D-SPT on neurons. No significant improvement was obtained using this technique in our experimental condition. Indeed, the main technical challenge in SPT on curved membranes is to use an acquisition frequency high enough to adequately follow the movements of the molecules without sacrificing positioning accuracy [28], and to adjust it as a function of the tube diameter.

SPT is a valuable technique that can be used not only on planar membranes but also on curved geometries, provided that geometrical effects are carefully taken into account. At the same time, SPT provides useful information about membrane shape and size at the length-scales below the diffraction limit of conventional microscopy (∼250 nm). The method has some similarity with PALM (photoactivable localization microscopy) in that it relies on the image reconstruction from a collection of single-molecule observations [29].

## Supporting Information

Figure S1 **Simulation of trajectories.** A) Example of a random walk simulation on the X–Y plane (*D* = 0.02 µm^2^/s, length of the trajectory: 200 points). The color of the trajectory changes upon time, starting with dark blue color and finishing with dark red color. B) Cylindrical trajectory (trajectory on the surface of a cylinder) obtained by enveloping a cylinder of 200 nm in diameter (broken line) with the simulated trajectory. C–D: The cylindrical trajectory was projected to the X–Y plane to obtain the projected trajectory.
(TIF)

Figure S2 **Construction of the calibration curve for 3D SPT.** *A*) Fluorescent beads were dried on a coverslip. Width of fluorescent beads spots in X (*black squares*) and in Y (*red circles*) vs. the position in Z of the coverslip (1 pixel = 110 nm). The microscope stage moved up with 10 nm steps. The panels below show images of beads at the indicated positions in Z (*arrows*). *B)* The mean ratio of the widths in X (*w_x_*) and Y (*w_y_*) was calculated for each position in Z. The calibration curve shows that the position in Z can be calculated in a ∼400 nm range.
(TIF)

Figure S3
**Effect of geometry and acquisition frequency on diffusion measurements on cylindrical structures.** *A*) Ratio of *D* calculated on projected trajectories (*D_proj_*) to *D* calculated on trajectories on cylindrical surfaces (*D_cyl_*) as a function of the diameter of the cylinder. The time between points *dt* was 15 ms. Each curve represents the mean ± SD values for 50 trajectories simulated to have the indicated diffusivities (0.001 to 1 µm^2^/s). *B*) The mean ratio *D_cyl_ / D_actual_* as a function of the dimensionless parameter (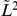) incorporating the diffusion coefficient (*D_actual_*), the image acquisition interval (*dt*) and the cylinder diameter (Ø). *C*–*E*) Ratios of *D* to the real diffusion constant of the original trajectory in the plane (*D_actual_*) calculated on trajectories constructed with different *dt* (C: 5 ms, D: 50 ms and E: 100 ms) on cylindrical surfaces (*A1,B1,C1; D_cyl_*) or projected (*A2,B2,C2; D_proj_*), as a function of the diameter of the cylinder. Each curve represents the mean ± SD values for 50 trajectories simulated to have the indicated diffusivities (0.001 to 1 µm^2^/s).(TIF)

Figure S4 **Measurement of the diameter of tubular structures from SPT data.** *A)* Diameter of artificial tubes from the limiting values of transversal MSD versus the diameter obtained by deconvolution. The *straight line* is a bisector where the two diameters are equal, which it is shown to emphasize the deviation from exact correspondence of the two estimates. *B)* Example of the transverse distribution of the QD positions on a neurite, extracted from a series of 1000 images (the positions are depicted in (*C)*). The distribution (*dashed lines and points in blue*) was fit by the convolution (*red line*). *C)* An example of QD positions on a neurite, extracted from a series of 1000 images. On the right, the rectangle used to measure the diameter of the neurite. Straight lines were drawn by eye enveloping the positions of QD. Several measurements were done on the same neurite, drawing the lines containing all the positions of QD or passing through the majority of positions at the border. Measurements were done transversally to the first line drawn (*blue*) at different places (exemplified by the *black lines* and *arrows*). The diameter was calculated as the mean ± SD of all the measurements (at least four). The obtained diameter and its error was comparable to the one measured in (*B*). *D*) Comparison of the tube diameters obtained from the shape like in *(C)* with the ones calculated based on the transversal MSD (mean ± SD for both). The *straight line* is a bisector where the two diameters are equal.
(TIF)

Movie S1 **Image sequence showing movements of quantum dot-labelled lipid on a lipid tube (right) pulled from a giant vesicle (left).** The synthetic lipid membrane was composed of sphingomyelin and cholesterol in 1∶1 molar ratio with 0.01 mol% of biotin-PEG2000-phosphatidylethanolamine as an anchor for streptavidin-coated QDs. The acquisition interval between frames was 15.7 ms, corresponding to slow-motion deceleration of around 2× in the movie. The scale bar is 5 µm.
(AVI)

Movie S2 **Diffusion of a QD bound to GFP-GPI on a neurite.** The QD (red) was tracked for 4s at 66 Hz (movie shown at half the real speed). The resulting trajectory (white) is shown on top of the fluorescence image of GFP-GPI. The pixel size is 167 nm.
(AVI)
