# Supplementary figures and images for: Lateral Diffusion on Tubular Membranes: Quantification of Measurements Bias
Source: PLoS One. 2011 Sep 29;6(9):e25731. doi: 10.1371/journal.pone.0025731 (PMC3183067; doi:10.1371/journal.pone.0025731)

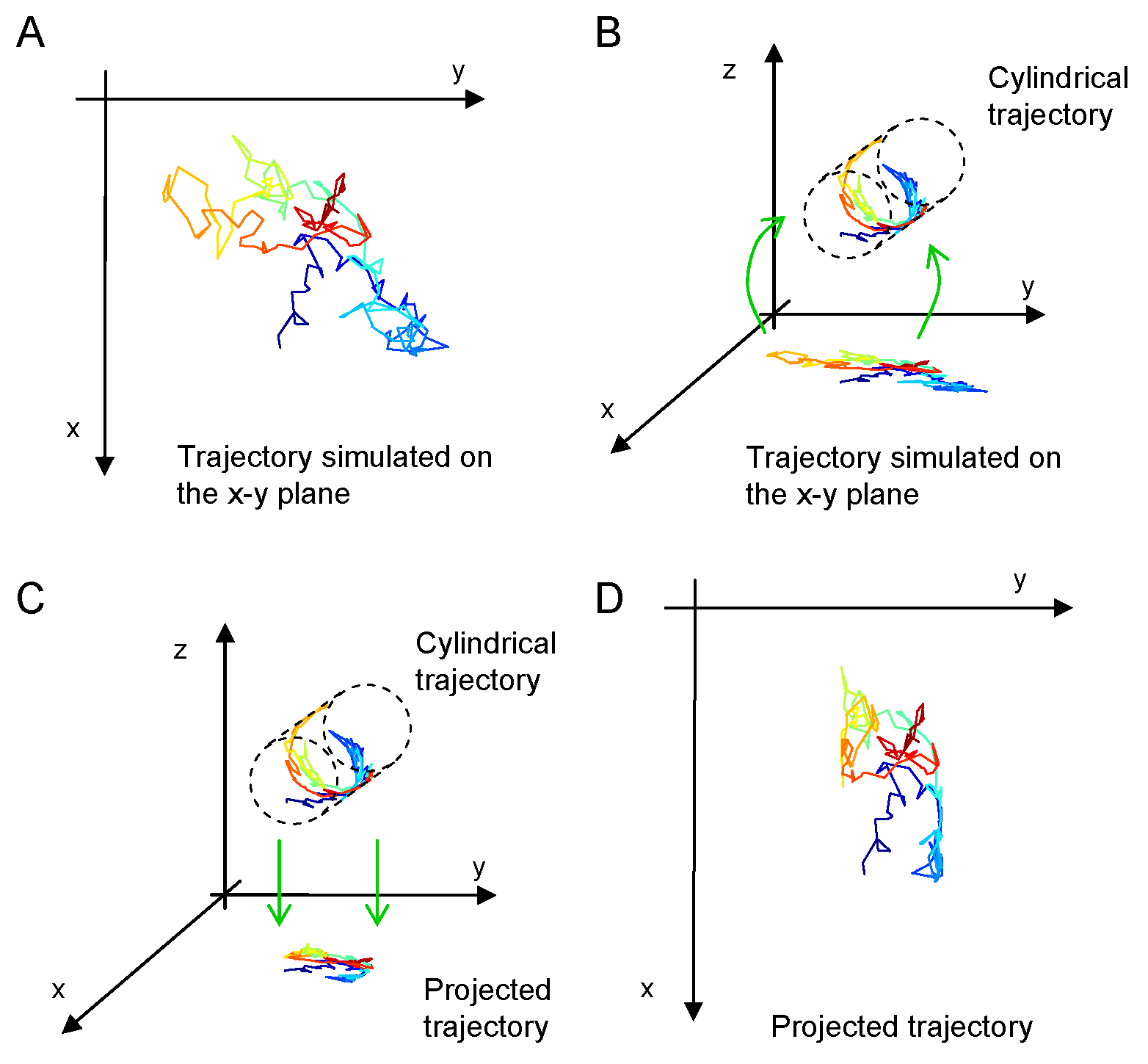

Supplement: Figure S1 — Simulation of trajectories. A) Example of a random walk simulation on the X–Y plane (D = 0.02 µm2/s, length of the trajectory: 200 points). The color of the trajectory changes upon time, starting with dark blue color and finishing with dark red color. B) Cylindrical trajectory (trajectory on the surface of a cylinder) obtained by enveloping a cylinder of 200 nm in diameter (broken line) with the simulated trajectory. C–D: The cylindrical trajectory was projected to the X–Y plane to obtain the projected trajectory. (TIF) [file pone.0025731.s001.tif]

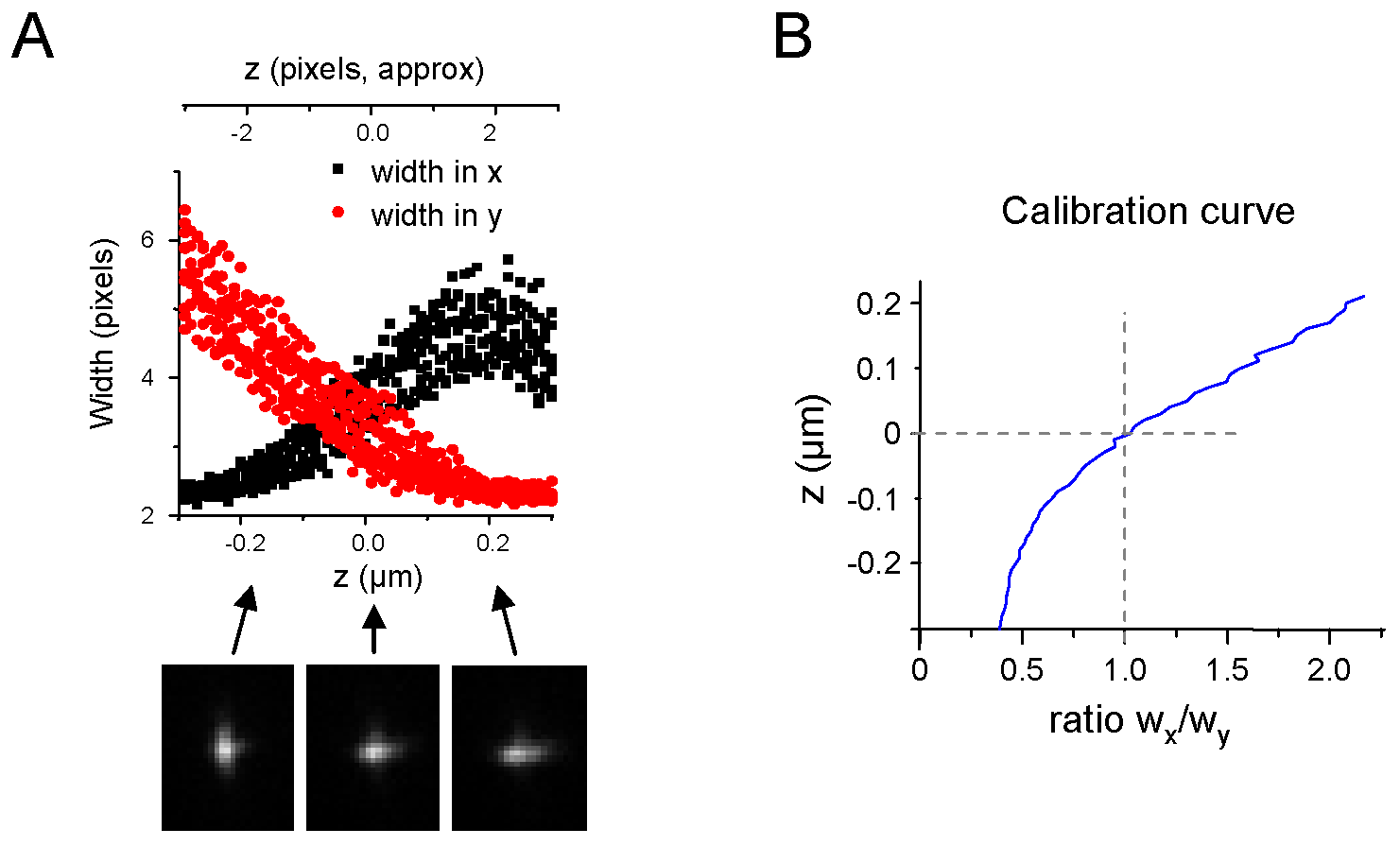

Supplement: Figure S2 — Construction of the calibration curve for 3D SPT. A) Fluorescent beads were dried on a coverslip. Width of fluorescent beads spots in X (black squares) and in Y (red circles) vs. the position in Z of the coverslip (1 pixel = 110 nm). The microscope stage moved up with 10 nm steps. The panels below show images of beads at the indicated positions in Z (arrows). B) The mean ratio of the widths in X (wx) and Y (wy) was calculated for each position in Z. The calibration curve shows that the position in Z can be calculated in a ∼400 nm range. (TIF) [file pone.0025731.s002.tif]

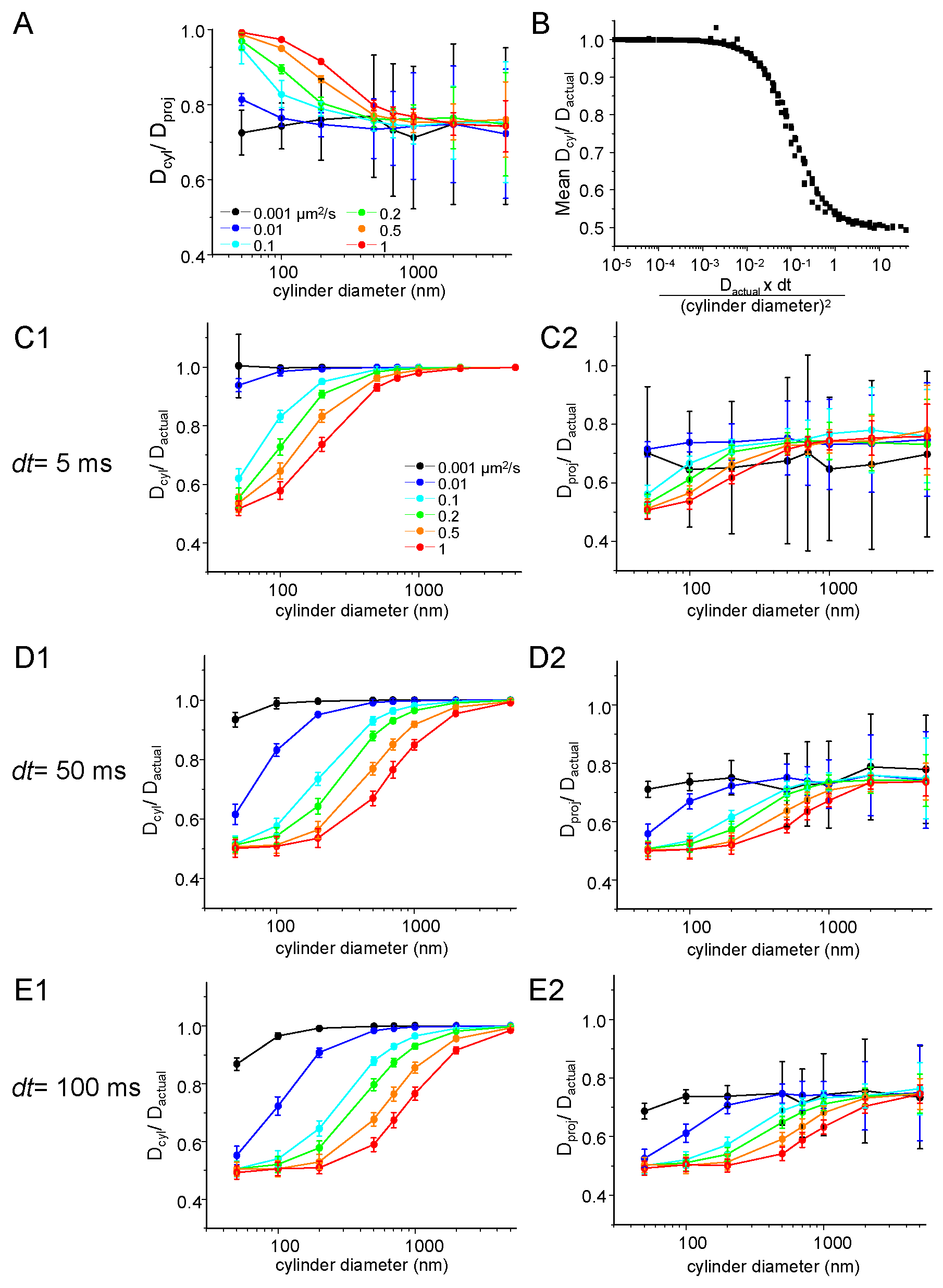

Supplement: Figure S3 — Effect of geometry and acquisition frequency on diffusion measurements on cylindrical structures. A) Ratio of D calculated on projected trajectories (Dproj) to D calculated on trajectories on cylindrical surfaces (Dcyl) as a function of the diameter of the cylinder. The time between points dt was 15 ms. Each curve represents the mean ± SD values for 50 trajectories simulated to have the indicated diffusivities (0.001 to 1 µm2/s). B) The mean ratio Dcyl / Dactual as a function of the dimensionless parameter () incorporating the diffusion coefficient (Dactual), the image acquisition interval (dt) and the cylinder diameter (Ø). C–E) Ratios of D to the real diffusion constant of the original trajectory in the plane (Dactual) calculated on trajectories constructed with different dt (C: 5 ms, D: 50 ms and E: 100 ms) on cylindrical surfaces (A1,B1,C1; Dcyl) or projected (A2,B2,C2; Dproj), as a function of the diameter of the cylinder. Each curve represents the mean ± SD values for 50 trajectories simulated to have the indicated diffusivities (0.001 to 1 µm2/s). (TIF) [file pone.0025731.s003.tif]

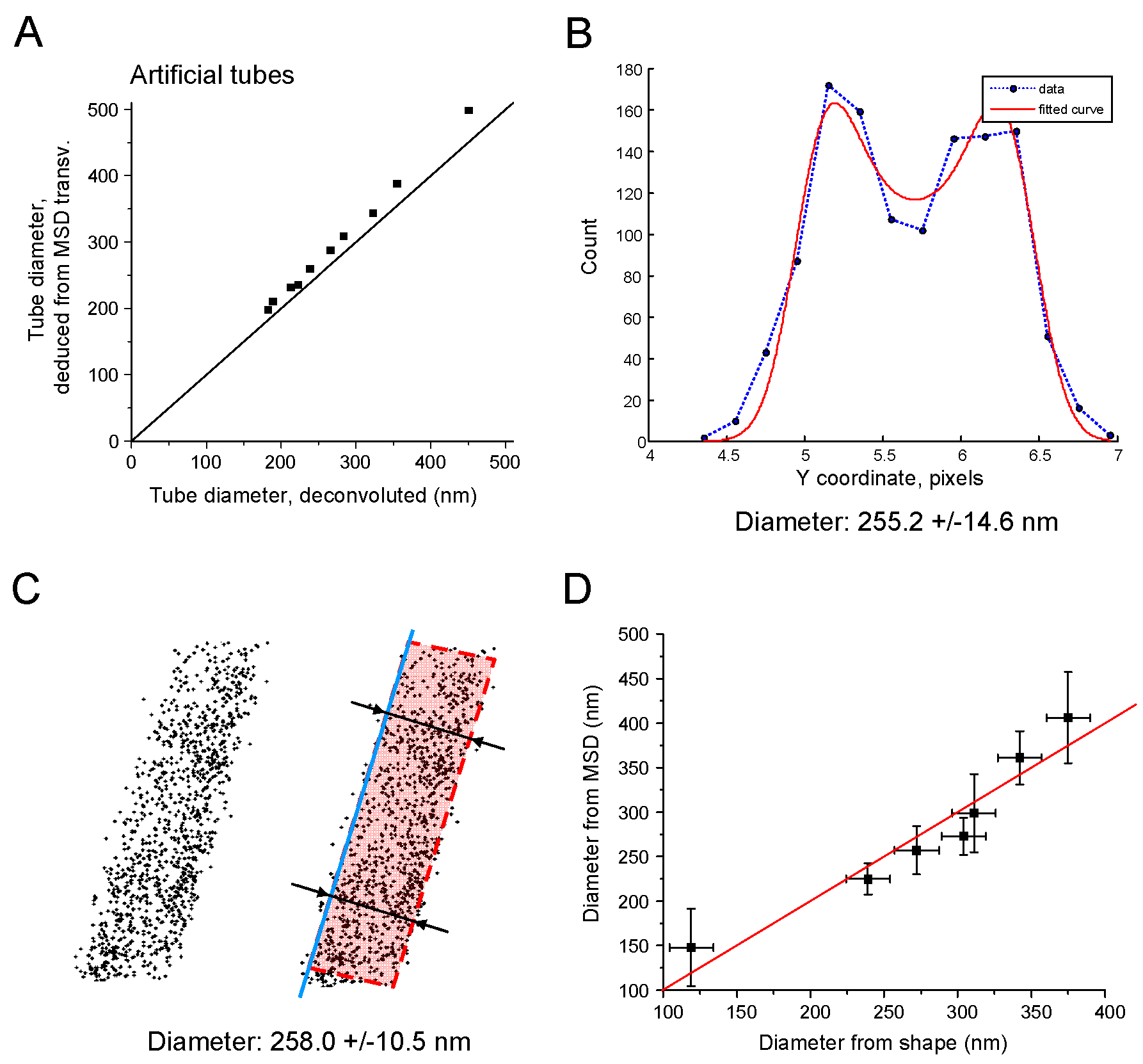

Supplement: Figure S4 — Measurement of the diameter of tubular structures from SPT data. A) Diameter of artificial tubes from the limiting values of transversal MSD versus the diameter obtained by deconvolution. The straight line is a bisector where the two diameters are equal, which it is shown to emphasize the deviation from exact correspondence of the two estimates. B) Example of the transverse distribution of the QD positions on a neurite, extracted from a series of 1000 images (the positions are depicted in (C)). The distribution (dashed lines and points in blue) was fit by the convolution (red line). C) An example of QD positions on a neurite, extracted from a series of 1000 images. On the right, the rectangle used to measure the diameter of the neurite. Straight lines were drawn by eye enveloping the positions of QD. Several measurements were done on the same neurite, drawing the lines containing all the positions of QD or passing through the majority of positions at the border. Measurements were done transversally to the first line drawn (blue) at different places (exemplified by the black lines and arrows). The diameter was calculated as the mean ± SD of all the measurements (at least four). The obtained diameter and its error was comparable to the one measured in (B). D) Comparison of the tube diameters obtained from the shape like in (C) with the ones calculated based on the transversal MSD (mean ± SD for both). The straight line is a bisector where the two diameters are equal. (TIF) [file pone.0025731.s004.tif]
